# Autonomic entrainment to music structure in Verdi opera

**DOI:** 10.1093/ehjimp/qyag025

**Published:** 2026-02-10

**Authors:** Natalia Cotic, Vanessa Pope, Pier D Lambiase, Elaine Chew

**Affiliations:** School of Biomedical Engineering & Imaging Sciences, King's College London, Strand, London WC2R 2LS, UK; School of Biomedical Engineering & Imaging Sciences, King's College London, Strand, London WC2R 2LS, UK; Department of Engineering, King's College London, Strand, London WC2R 2LS, UK; University College London and Barts Heart Centre, West Smithfield, London EC1A 7BE, UK; School of Biomedical Engineering & Imaging Sciences, King's College London, Strand, London WC2R 2LS, UK; Department of Engineering, King's College London, Strand, London WC2R 2LS, UK

**Keywords:** autonomic nervous system, cardiorespiratory entrainment, musical phrase arc boundaries, time-frequency analysis

## Abstract

**Aims:**

Music can influence bodily rhythms, offering a powerful way of modulating autonomic physiology. Entrainment between music structure and physiologic responses provides a potential mechanism for this effect. This study examined (1) whether autonomic entrainment is driven primarily by objective music structure or by listeners’ subjective perception of music boundaries and (2) how structural and dynamic features of opera shape entrainment.

**Methods and results:**

Twenty-four participants (12 choristers, 12 non-choristers) listened to two Verdi excerpts while respiration, RR intervals, and continuous blood pressure were recorded. Entrainment between music features (loudness and tempo) and physiology was assessed using time-frequency coherence, revealing significant intra- and inter-individual coupling, strongest during Nabucco. Surrogate testing confirmed that these effects were linked to music structure rather than incidental physiologic fluctuations. Music boundaries were modelled as Gaussian envelopes derived from participant-defined (subjective) annotations and trained-annotator (objective) annotations. Objective boundary envelopes aligned more closely with physiologic envelopes than subjective annotations across signals. Subjective boundary performance improved when restricted to higher-strength annotations and when closely matching music structural changes. Choristers’ boundary annotations were more consistent within the group, but overall physiology–music entrainment strength was similar between choristers and non-choristers.

**Conclusion:**

Music structure plays a key role in shaping autonomic entrainment. Autonomic entrainment during music listening is most consistently explained by music structure changes, particularly well-defined and salient boundaries (objective annotations), rather than by an individual's perception of structure changes (subjective annotations). These findings support a scalable framework for music-based interventions grounded in extractable structural features rather than personalized perception.

## Graphical Abstract

**Graphical Abstract:**
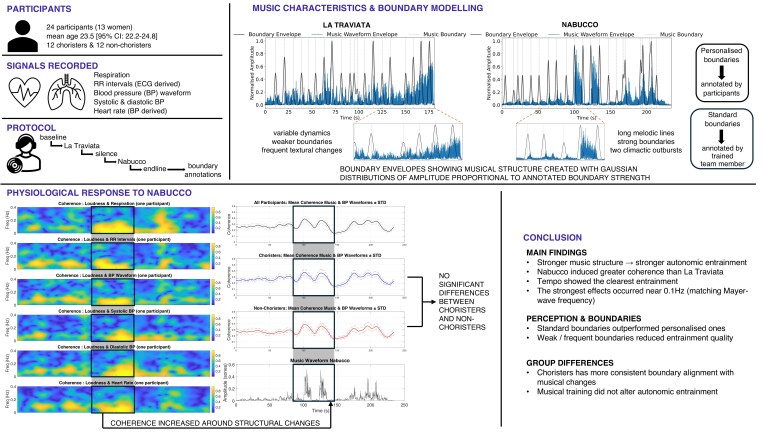

## Introduction

Cardiovascular disease remains the leading cause of mortality worldwide,^1^ prompting increasing interest in non-pharmacologic and scalable interventions for autonomic regulation. Among these, music listening has shown considerable promise for influencing heart rate, respiration, and blood pressure in both clinical and everyday settings.^2–8^ Yet the mechanisms underlying music’s physiologic impact remain only partially understood, limiting its potential for scalable therapeutic applications.

A landmark study by Bernardi *et al*.^9^ demonstrated that autonomic variables can synchronize with temporal features of music, particularly vocal music by Verdi, through a process of physiologic entrainment. In their findings, dynamic cardiovascular and respiratory signals appeared to ‘mirror’ fluctuations in the music’s amplitude envelope, with some of the strongest effects occurring at frequencies ∼0.01 Hz, aligned with Mayer waves—oscillatory rhythms tied to baroreflex function. Notably, these entrainment effects emerged regardless of participants’ emotion responses or music background, suggesting that structural cues in music may engage subconscious autonomic processes.^9,10^

Recent advances in music information research (MIR) and signal analysis now enable a more precise investigation into how music’s structure may influence the body. Music expression often unfolds to reveal arc-like patterns in features such as loudness and tempo that shape perceived phrases.^11–14^ These arc-like changes, called phrase arcs, are thought to play a crucial role in guiding listener attention and engagement.^15–17^ However, prior studies have not addressed a fundamental question: how do listeners’ perceived structural segmentation of music (phrase arc boundaries^11–14^) shape physiologic entrainment?^18^

Here, we extend Bernardi *et al*.’s findings by introducing a novel boundary-driven envelope modelling framework, where music’s dynamic structure is represented as a series of Gaussian curves centred on perceptually salient segmentation points. These partitioning points mark the phrase arc boundaries, which are believed to influence responses to music.^15–17^ These boundaries were annotated by participants themselves (subjective) or by a member of the team trained in phrase arc identification (objective), allowing for direct comparison of entrainment effects between canonical and subjective perceptions of music structures.

Comparing physiologic envelopes to the subjective and objective boundary envelopes will test whether entrainment follows the listener’s personal perception of phrase arc boundaries rather than a standard benchmark segmentation. Our design directly looks into these factors and tests how strongly individual perception plays a role in physiologic reactions to music, which will inform the choosing of music for targeted responses in digital music therapeutics. Perceived structure and aesthetic preference are distinct but interact in ways that matter for entrainment and for clinical translation. First, listeners’ perception could affect when and how they react to the music stimulus.^19–21^ Second, individual preference could modulate the degree of arousal, which would influence the response.^22,23^ Analysing how music preference and perception affect coherence and entrainment will inform the framework for using music in personalized medicine. Prior research suggests that these factors do not play such an important role in how someone reacts to the music stimulus, but instead, the reaction is driven subconsciously by the autonomic nervous system responding to basic music structures.^24–26^

The current study had three primary goals: (1) replicate and validate Bernardi’s original results using current MIR and time–frequency coherence methods, (2) investigate whether objective boundaries induce stronger autonomic entrainment than subjective ones, and (3) examine how boundary strength and music training (chorister vs. non-chorister status) influence the degree of entrainment. By addressing these questions, our findings aim to clarify the relationship between music perception and physiologic modulation, with implications for personalised and group-based music therapies.

## Methods

### Participants and study protocol

Participants were chosen to match the Bernardi study demographics. 24 participants (13 women, mean age 23.5 [95% CI: 22.2–24.8]) took part in data collection. 12 were choristers, while the other 12 were not musically trained. As mentioned, the two tracks that induced increased coherence and entrainment in^9^ were chosen for this study. Giuseppe Verdi’s arias from Nabucco and La Traviata have rhythmic phrases of 10 s/0.1 Hz and coincide with the Mayer waves in the body. The tracks were from the same recordings and of the same duration as the ones described in the Bernardi study.

A study session was comprised of 5 min of initial baseline silence followed by ‘Libiam nei lieti calici’ from La Traviata performed at the Royal Opera House (Decca 1995), followed by 2 min of silence, then ‘Va Pensiero’ from Nabucco performed by Berlin State Opera Chorus (DG 1984) and ended with another 5 min of endline silence. The music was played through the HeartFM app^27^ which also allowed for the synchronization and recording of the physiologic data. Participants listened to the music through a set of wireless Bluetooth headphones kept at the same system volume to ensure consistency between participants.

The initial and end silences allowed for the analysis to investigate changes in physiologic interactions before and after the music stimulus. The silence between the tracks ensured any lingering effect from the previous track did not influence the subsequent one. Demographic data was recorded from each participant and their music knowledge was quantified using a reduced Goldsmith’s Music Sophistication Index questionnaire.

Following the listening session, the recording equipment was removed, and participants were asked to annotate perceived phrase boundaries. Each person was told that phrases represent clear sections that have a build-up and conclusion, and a boundary denotes a new phrase beginning.^11,12,14–17^ Participants were asked to annotate these boundaries based on their perception. Moreover, they were given the option to include the strength of the boundary by using different keys for the markings (1: weakest, 4: strongest). The annotations were done on RumiNote, a web-platform built by the team specifically for the purpose of the study. RumiNote allows for the upload of audio and the creation and download of annotations.

### Music section and phrase boundaries

The two Verdi opera selections highlighted for strong coherence results in,^9^ Nabucco and La Traviata, were used for this study. Objective annotations of sections and phrase boundaries were done by a member of the team trained in phrase arc identification.

**Figure 1:**
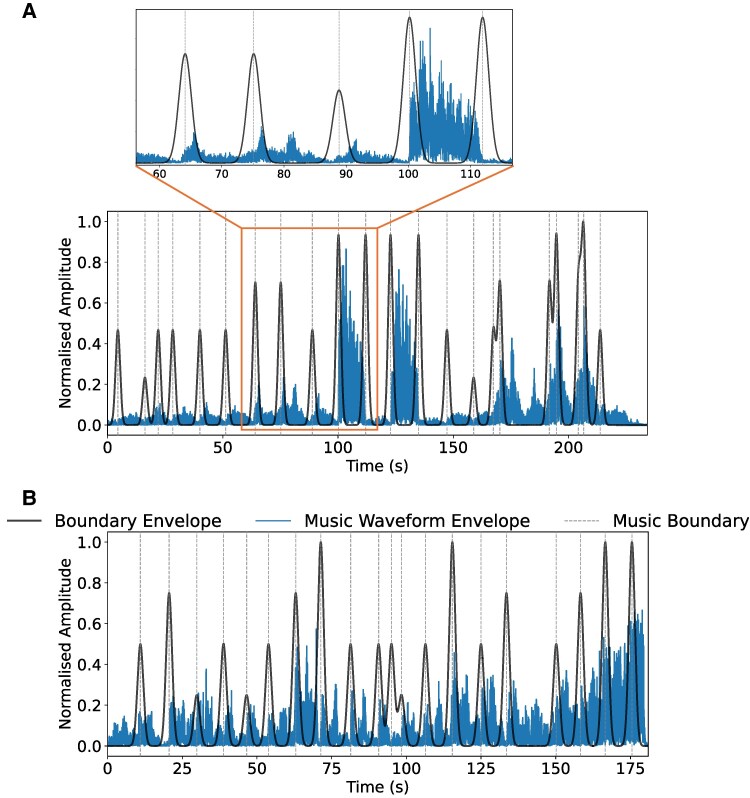
Visualization of music waveform envelopes for Nabucco and La Traviata with boundaries placed between music sections and phrases. The resulting boundary envelopes delineate the perceived music phrase arcs, which create structural segmentations in the music. (*A*) Nabucco shows well-defined changes which are visible in the audio waveform, resulting in high magnitude boundaries separating the music sections. Zoom in shows how the audio corresponds to the annotated boundaries. (*B*) La Traviata has more variability throughout its waveform, making the music sections less distinct and resulting in weaker peaks in the music boundary envelope.

‘Va, pensiero’ from Nabucco is a choral piece with sustained melodies and smooth phrase transitions interrupted by two dramatic outbursts each lasting approximately 10 s. The quieter sustained parts and two loud sections are visible in the audio waveform, shown in blue in *Figure 1A*. Section and phrase arc boundaries are identified and marked by a team member. The boundaries, shown as vertical dotted grey lines, are transformed to continuous Gaussian envelopes with peaks reflecting the boundaries’ strengths. Note that the peaks in the boundary envelope occur at approximately 10-s intervals as shown in the zoomed-in image. The contrasting loud section around 100 to 110 s is delimited on either side by high amplitude (strong) boundary envelope peaks. Such stable and predictable interval between boundaries has been found to be conducive to synchronization between music and physiology and to improve entrainment.^28^

The other piece, ‘Libiam nei lieti calici’ from La Traviata, is a lively waltz with alternating textures comprising of solo passages, interjections from the chorus, and a central duet between the two principal singers. The starts and ends of these sections form sectional boundaries. The music has frequent changes in instrumentation, vocal texture, and dynamics, contributing to boundaries of varying strengths as shown in *Figure 1B*. Compared to the Nabucco excerpt, the thresholds between sections are less well defined, having lower amplitude peaks. The high degree of variability in this piece, while musically engaging, may pose more challenges for physiologic entrainment compared to music that is more predictable.

By comparing and contrasting how participants’ physiology entrained to these distinct opera excerpts, we will determine which music features enhance entrainment and clarify how perceived music structure impacts physiologic response.

### Data collection

Autonomic physiological signals—electrocardiographic traces (ECGs), respiration, and continuous blood pressure—were recorded during the first part of the session, comprising of music listening and silences. ECGs showing the electrical activity of the heart were recorded using a Polar H10 sensor capturing data at a sampling frequency of 1000 Hz. The RR intervals (time between adjacent R peaks on the ECG) were extracted by the Polar software which automatically down samples them to 130 Hz for export. Manual checks and corrections were performed to ensure all R peak markings and the resultant RR intervals were correct. Respiration data was collected using a BIOPAC respiratory effort transducer and belt that recorded the stretch of the sensor, showing the degree of expansion of the chest, at a rate of 13 Hz. This signal was corrected through a rolling average which removed the baseline drift and a 4th order Butterworth filter with a 1 Hz cut-off frequency for the removal of noise from the apparatus. The continuous blood pressure waveform, individual systolic and diastolic components, and the heart rate were recorded using a CNAP500 monitor comprising of arm and finger sensor cuffs. Data from the CNAP did not require any pre-analysis processing. The data pre-processing steps ensured the quality of the physiologic data without affecting their morphology.

The HeartFM mobile app^27^ was used to play music to the participants, and also to record and synchronize the data. The respiration, ECG, and extracted RR intervals were wirelessly passed from the sensors to the device with the HeartFM app running. Data was saved with timestamps of the music track start and end times. The data from the CNAP500 monitor was manually synchronized to the others by asking the participants to give a squeeze to the cuffed fingers during a countdown to the beginning of the recording session. The squeeze created a spike in the signal, which was used as a time reference and showed where to trim the data.

The music track timestamps from the app were then used to segment all the signals into corresponding files for each of the two recordings played with baseline and endline, and silence between the recordings. The beats of the two music tracks were manually tapped by a musician member of the team using SonicVisualiser.^29^ The beat markings and audio files were passed to the MATLAB MA Toolbox to compute the perceived loudness of the music in sones. The tempo in beats per minute was calculated from the intervals between beat markings.

The time axis of each file was offset to zero and all physiologic signals were placed on a common time axis and interpolated to 4 Hz to match the format of the Bernardi^9^ data. The music envelopes were computed by applying the Hilbert transform to the music audio. This created a smooth, frequency-independent signal that preserved timings and dynamic changes in the music and permitted comparison with the physiologic and music feature signals.

### Coherence analysis

#### Smoothed Pseudo–Wigner–Ville distribution

Following the pre-processing steps, the first analysis method involved calculating the coherence between signals to validate the findings of.^9^ To quantify the dynamic coupling between physiologic signals and music features, we computed time–frequency coherence using the smoothed pseudo–Wigner–Ville distribution (SPWVD).^30,31^ This method is well suited to identifying cardiovascular oscillations, such as Mayer waves (∼0.01 Hz), while providing high temporal and spectral resolution, and retaining the temporal localization of coherence peaks occurring with the music features. Coherence matrices were computed consistently across participants. This was facilitated by the interpolation applied to all signals, which brought them to a common time axis with an identical sampling frequency. (Full SPWVD/TFC equations, kernel definition, and parameter selection details moved to Supplementary Methods  *S1*.)

#### Statistical analysis using surrogate testing

Statistical significance was assessed using within-subject surrogate testing. Surrogate data provided a participant-specific null distribution. Observed music-physiologic coherence values were compared to these distributions. The surrogate datasets were created by concatenating an individual’s physiologic data for the entire recording, excluding the track being analysed.^32,33^ The analysis was re-run with segments from the surrogate dataset and the analysed track's music signal. This method preserved each participant's unique physiologic response pattern while disrupting the time alignment with the track being analysed.

For each participant, we computed the difference between the coherence observed during a piece and the participant's surrogate mean. A *t*-test on these within-subject differences assessed whether observed coherence differed from the surrogate one. This allowed us to ascertain whether or not the trend observed in the real values could be attributed to the music to which the participant was listening. Bonferroni correction was applied to account for having 6 tests per music feature of a track, resulting in the new *P*-value being 0.008.

To identify if there is a clear influence on the coherence due to the music, we compared the music envelope (Hilbert transform of the audio signal) with the mean coherence envelope of all participants. The visual representation of this comparison is seen in *Figure 3*, which is described further in the Results section.

### Subjective vs. objective boundary comparison

The other main analysis method involved the use of music phrase arc boundaries. The subjective sets of boundaries were taken from each participant's annotations in RumiNote. Objective boundaries were made by a member of the team and aimed to mark changes in the music structure. Using these annotations, the phrase boundary envelope was computed for a track. A Gaussian of fixed width (sigma = 1 s) was centred on the timestamp of a boundary annotation. The strength of the marker (1: weakest, 4: strongest) was used to scale the amplitude of the curve. The resulting Gaussian curve was added to the envelope, allowing multiple boundaries to overlap and build up a continuous boundary envelope.

The Gaussian curve generated from the annotated boundaries represented the phrase boundaries in the music delimited by the annotations. A curve was generated for each of the subjectively perceived music structure boundaries annotated by the participants for the two tracks. For the objective (trained-annotator-marked) boundaries, the phrase arc envelope was computed once for each track across all participants. The envelopes were then compared with people’s physiology.

The comparisons between the physiology and the two types of boundary envelopes were carried out separately. This showed whether an individual’s physiologic changes were more synchronized with (1) their personal perception of the music structure changes, or (2) objective music structure changes. The evaluation of the entrainment was done through Earth Mover’s Distance (EMD) using the Wasserstein function in Python. EMD calculates the cost of transforming one distribution into another and generates a value indicative of the similarity between the two signal arrays given. A smaller EMD value shows increased similarity as it costs less to match the distributions. Other metrics, such as Dynamic Time Warping and Euclidean Distance, were also tested; however, they were either too computationally expensive or prone to producing outlier values.

**Figure 2:**
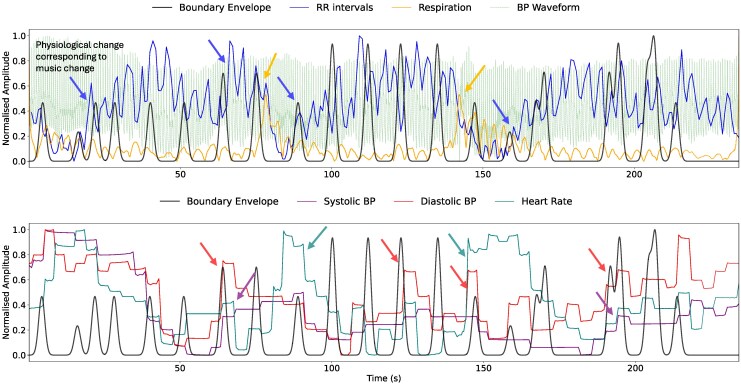
Example of boundary and physiologic signal envelopes utilized in the analysis. Changes in physiology occurring around time of boundaries (peaks in black line) marked with arrows matching the colour of the corresponding physiologic signal envelope.

To compare boundary annotations, the physiologic data did not require interpolation, so the signals were only cleaned. To ensure that the EMD results were not skewed due to high variability in the data arrays, the envelopes of the pre-processed signals were computed using the Hilbert transform and can be seen in *Figure 2*. This transform produces smooth, continuous, and frequency-independent envelopes without removing the morphological information that can be derived from them. These physiologic envelopes were more suitable for comparison with the phrase arc envelopes. The EMD calculated with the two sets of data were then compared using paired *t*-tests to see the boundaries with which people were more synchronised.

## Results

This study’s central contribution is a boundary-driven framework for modelling music structure and testing whether autonomic responses align more closely with objective, trained-annotator boundaries or with subjective, participant-perceived boundaries. We first present time–frequency coherence results that validate and contextualize these findings relative to Bernardi *et al*. followed by our novel findings on the boundary-envelope analysis (Earth Mover’s Distance comparisons).

### Coherence analysis

**Figure 3:**
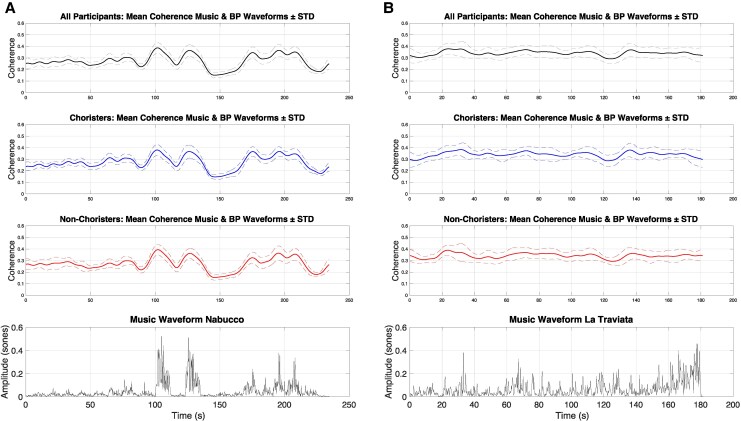
Mean coherence between loudness and blood pressure during music listening for all participants and grouped by music training. Coherence peaks are seen at the same time as music structure changes in the audio waveform. (*A*) Nabucco shows well-defined changes in the music waveform, resulting in stronger coherence during those sections. (*B*) La Traviata has more variability throughout its music waveform, with a weaker coherence response.

**Figure 4:**
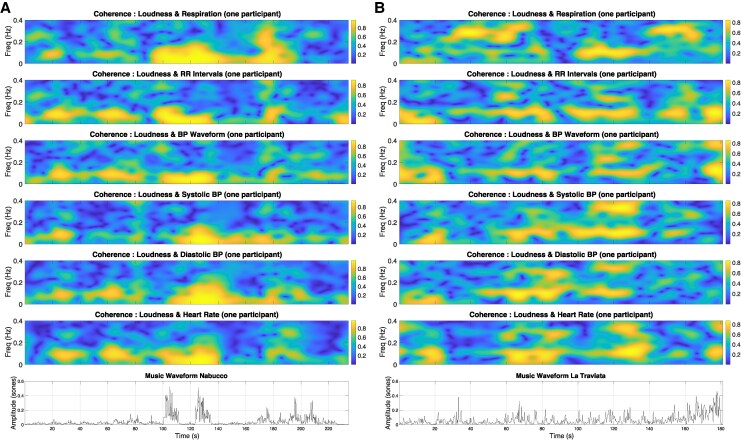
Coherence between loudness and physiologic signals during music listening for a participant. Coherence peaks are seen at the same time as well-defined music structure changes/peaks in the music waveform (bottom subplot). (*A*) Nabucco shows increased coherence with well-defined sections, which also match the Mayer wave period in their duration (∼10 s). (*B*) La Traviata has more variability throughout, but exhibits increased coherence around the more well-defined regions of change.

We validated and confirmed that coherence was influenced by music structure changes, corroborating the findings in the original study. Coherence peaks occurred around salient music transitions in both opera excerpts (*Figures 3, 4*), with stronger and more sustained responses during Nabucco.

Just as in,^9^ blood pressure components had strong entrainment with the music features of Nabucco, especially around the fortissimi (high amplitudes) in the music waveform. The significance was evaluated through the comparison of the results with surrogate data, which was individually generated to test against the null hypothesis. Looking at the significance of the results shown in *Table 1*, the tempo for Nabucco showed strong coherence with the blood pressure (BP) components: systolic BP (*P* = 0.0003) and diastolic BP (*P* = 0.0009). Nabucco's loudness also synchronized with the systolic BP (*P* = 0.006). For La Traviata, the tempo had increased coherence with the RR intervals (time between adjacent R peaks on the ECG) (*P* = 0.004). *Table 2*, shows that all participants had increased coherence between the RR intervals and the diastolic BP during Nabucco compared to silence (*P* = 0.05).

**Table 1:** Real vs. surrogate mean coherence during music for all participants

Track	Music signal	Physiological signal	Coherence actual	Coherence surrogate	*t*-statistic	*P*-value
Nabucco	Tempo	Systolic BP	0.23	0.20	4.25	0.0003*
Nabucco	Tempo	Diastolic BP	0.23	0.21	3.82	0.0009*
La Traviata	Tempo	RR intervals	0.28	0.24	3.24	0.004*
Nabucco	Loudness	Systolic BP	0.23	0.21	3.00	0.006*

Nabucco's tempo shows most significance.

^*^Significant after Bonferroni correction.

**Table 2:** Coherence during Nabucco vs. silence for all participants

Pair	Coherence Nabucco	Coherence silence	t-statistic	*P*-value
RR int diastolic BP	0.31	0.28	2.03	0.05*
Respiration BP waveform	0.22	0.21	1.92	0.06
RR int systolic BP	0.29	0.26	1.88	0.07

Coherence between physiologic signals changes significantly between RR intervals and diastolic BP, other pairs approach significance.

^*^Significant after Bonferroni correction.

Increased coherence was observed between individuals’ signals during music listening, with the observed effect being stronger for Nabucco than La Traviata. Accounting for multiple tests with Bonferroni correction, the new critical *P*-value became 0.008. Subsequently, only the respiration and RR interval coherence remained significant for La Traviata; whereas, for Nabucco, all but the heart rate signals showed significantly increased inter-individual coherence during music listening (see *Table 3*).

**Table 3:** Real vs. surrogate results for inter-individual coherence comparisons

Track	Signal	Music coherence	Surrogate coherence	t-statistic	*P*-value
LT	Respiration	0.20	0.19	6.05	4.51E−09*
LT	BP waveform	0.32	0.32	2.85	4.64E−03*
LT	RR intervals	0.26	0.23	9.72	2.20E−19*
LT	Systolic BP	0.25	0.24	2.53	1.21E−02
LT	Diastolic BP	0.25	0.24	2.95	3.44E−03*
LT	Heart rate	0.23	0.23	0.13	8.92E−01
Nab	Respiration	0.20	0.18	4.58	6.95E−06*
Nab	BP waveform	0.33	0.32	6.34	9.35E−10*
Nab	RR intervals	0.24	0.22	5.27	2.76E−07*
Nab	Systolic BP	0.25	0.22	9.20	9.30E−18*
Nab	Diastolic BP	0.24	0.23	4.42	1.41E−05*
Nab	Heart Rate	0.22	0.22	1.26	2.09E−01

Nabucco leads to stronger real vs. surrogate coherence differences than La Traviata. LT: La Traviata, Nab: Nabucco, BP: blood pressure.

^*^Significant after Bonferroni correction.

Group comparisons (choristers vs. non-choristers) are exploratory, given the modest sample size and were not powered to detect small-to-moderate effects. Consistent with Bernardi *et al*., group-level coherence patterns were broadly similar for choristers and non-choristers (*Figure 3A* and *B*). In exploratory analyses, a small number of comparisons reached nominal significance; however, given limited power and multiple testing, these effects should be interpreted cautiously and not as evidence of robust training-related differences. Overall, the data suggest that the main entrainment signatures observed here are not strongly contingent on music training level.

### Boundary envelope analysis

The influence of perception was tested by comparing physiologic envelopes with boundary-based models of music structure. Subjective, participant-perceived boundaries were obtained from each participant’s RumiNote annotations, whereas objective, trained-annotator boundaries were annotated by a team member trained in phrase arc identification and applied across participants for each excerpt. Each boundary set was converted into a continuous boundary envelope by summing Gaussian curves centred on boundary timestamps, with amplitudes scaled by the annotated boundary strength from 1 (weakest) to 4 (strongest).

#### Objective boundaries align better than subjective boundaries with physiologic changes

The objective boundaries, set by a trained annotator, had better alignment with the changes in physiology compared to the subjective annotations. Increased similarity between the music and physiology envelopes was consistently seen for the objective annotations, indicating better alignment with the body's response. The EMD between the boundary envelope and the physiologic signals for each track is shown in *Table 4*. The greater effect of the objective boundaries was more strongly observed in Nabucco across all music-physiology pairs (*P* < 1.67E−04). For La Traviata, only one pair was still significant following Bonferroni correction (critical *P*-value = 0.008). Cohen's *d* (standardized measure of differences between sets) was also computed to assess the significance/effect size of the results. For La Traviata, the effect strength is medium, whereas for Nabucco it is more significant, showing large to very large differences between the subjective and objective perception of music structure boundaries.

**Table 4:** Statistical comparison between EMDs of subjective vs. objective boundary envelopes and physiologic signals

Track	Physiological Signal	EMD Subjective	EMD Objective	t-statistic	*P*-value	Cohen’s *d*
LT	RR intervals	17.13	14.22	1.33	1.96E−01	0.27
LT	Respiration	155.99	114.71	2.04	5.35E−02	0.42
LT	BP waveform	1330.14	925.84	2.27	3.32E−02	0.46
LT	Systolic BP	24.23	19.27	2.08	4.84E−02	0.43
LT	Diastolic BP	22.92	17.50	2.11	4.57E−02	0.43
LT	Heart rate	19.84	13.09	3.09	5.22E−03*	0.63
Nab	RR intervals	27.94	13.27	7.53	1.20E−07*	1.54
Nab	Respiration	293.82	170.39	5.93	4.79E−06*	1.21
Nab	BP waveform	2132.48	1031.05	6.56	1.09E−06*	1.33
Nab	Systolic BP	33.42	23.72	4.08	4.61E−04*	0.83
Nab	Diastolic BP	31.74	22.33	4.02	5.31E−04*	0.82
Nab	Heart rate	29.32	15.60	6.34	1.79E−06*	1.29

Trend shows that listeners synchronized better with the objective boundaries. The difference was less significant for LT once Bonferroni correction was applied. Results for Nab are significantly different throughout. LT: La Traviata, Nab: Nabucco, BP: blood pressure.

^*^Significant after Bonferroni correction.

#### Higher agreement between stronger subjective boundaries and objective boundaries

*Table 5* summarizes additional tests examining which aspects of subjective annotation drive the observed discrepancy relative to the objective envelopes. To test whether physiologic alignment is driven primarily by stronger boundaries, we repeated the EMD comparisons using only higher-strength subjective boundaries (strength 3–4). Removing low-strength boundaries reduced the difference between subjective and objective envelopes across most signal pairs (*Table 5*, 4th column vs. 3rd), indicating that weaker annotations contribute disproportionately to envelope variability and reduced physiologic relevance. For Nabucco, a subset of pairs remained significantly different after correction, suggesting that even the stronger subjective boundaries capture only part of the structure that is reflected in physiology. In general, filtering by strength reduced the discrepancy between the two boundary envelope types.

**Table 5:** *T*-test *P*-value when comparing the subjective boundary envelopes and the modified subjective envelopes (with low strength boundaries removed and only looking at sensible annotations) to the objective envelope results

Track	Physiological signal	Subjective *P*-value	Boundary removal *P*-value	Sensible *P*-value
LT	RR	1.96E−01	6.32E−01	8.74E−01
LT	Resp	5.35E−02	8.11E−01	3.82E−02
LT	Waveform	3.32E−02	8.95E−01	5.99E−01
LT	Systolic	4.84E−02	3.87E−01	8.22E−01
LT	Diastolic	4.57E−02	3.07E−01	4.92E−01
LT	HR	5.22E−03*	4.97E−01	9.24E−03
Nab	RR	1.20E−07*	2.02E−02	1.29E−04*
Nab	Resp	4.79E−06*	8.29E−02	5.20E−03*
Nab	Waveform	1.09E−06*	8.75E−03	3.03E−03*
Nab	Systolic	4.61E−04*	7.94E−04*	3.75E−02
Nab	Diastolic	5.31E−04*	1.19E−03*	1.27E−01
Nab	HR	1.80E−06*	9.96E−03	2.70E−03*

Decrease in significance for the modified subjective compared to the unprocessed subjective envelopes indicates that these modifications brought the entrainment results closer to the ones of the objective boundaries. They led to a performance improvement for the subjective annotations.

^*^Significant after Bonferroni correction.

#### Subjective annotations matching music structure changes performed better

We next analysed whether subjective boundary envelopes become more physiologically informative when participants segment the music at a granularity comparable to the objective annotations. Subjective markings varied widely (20–110 for La Traviata; 10–190 for Nabucco), whereas the objective boundary counts were 20 and 22, respectively. Restricting the analysis to participants who marked a moderate number of boundaries (15–30; *n* = 12) reduced the subjective-objective discrepancy across most pairs (*Table 5*, 5th column vs. 3rd), consistent with improved alignment when subjective segmentations are near salient structural changes. For Nabucco, several pairs remained significantly different, indicating that matching boundary density alone does not fully account for the stronger physiology–objective alignment.

#### Similar entrainment to objective boundary envelopes for choristers and non-choristers

No significant group differences were observed in physiology-boundary entrainment for choristers and non-choristers. Choristers’ boundary timing was more internally consistent than non-choristers’ (lower annotation variability), but this did not translate into systematically stronger physiologic alignment.

## Discussion

Our boundary analysis findings indicate that autonomic alignment tracks objective annotation, guided by music structure, more strongly than the subjective annotation, determined by idiosyncratic perception. The subjective boundary markings approach physiologic relevance when they converge on the same high-salience structural events.

### Music boundaries’ impact on entrainment

Objective boundary envelopes had better alignment with physiology compared to participants’ subjective ones. The objective boundaries indicated structural changes such as musical closure, tension-release, and phrase demarcation, making them more effective in matching autonomic variations than subjective segmentations. The subjective boundaries displayed greater inter-subject variability, and entrainment values between these boundary envelopes and physiology showed broader distributions, reducing their explanatory power. While individual interpretation has been shown to influence the response to the music^22,23^; the results could indicate that the objective changes in music features supersede personal interpretation of music structure changes when it comes to driving physiologic responses.

Annotations of weak boundaries introduced substantial variability in the subjective envelopes. Removing lower-strength markings (strength 1–2) reduced the discrepancy between subjective and objective envelopes, suggesting that low-salience annotations contribute disproportionately to noisy fluctuations rather than physiologically meaningful structure. Weak boundaries may nonetheless reflect sub-phrase segmentation or hierarchical groupings^34^; however, such fine-grained or frequent transitions may be insufficiently salient to elicit robust autonomic modulation.^35^

Participants whose subjective annotations more closely matched the objective boundary structures showed greater entrainment between their boundary envelopes and physiology. Restricting the analysis to participants with a modest number of markings reduced variability and revealed stronger alignment when the subjective annotations concurred with salient structural changes. Choristers were as likely as non-choristers to annotate close to the objective boundaries, indicating that sensitivity to musical structure does not require formal musical training. Although minor group-level differences were observed, these were not statistically significant.

Exploratory comparisons suggested that choristers’ boundary annotations were more internally consistent (lower timing variability) than non-choristers’ annotations, which could be indicative of choristers having a similar music knowledge base. However, this difference relates primarily to annotation consistency rather than to stronger physiologic alignment *per se*, and we did not observe clear evidence that music training produces systematically stronger physiology–boundary entrainment. Larger samples will be required to determine whether training plays a substantial role in segmentation reliability and whether such differences translate into measurable physiologic effects. Future work will aim to incorporate information on music training and personal taste from the reduced Goldsmith Music Sophistication Index questionnaire that all participants completed, allowing us to investigate whether these potentially confounding variables play a role in shaping the observed responses.

Importantly, this boundary envelope analysis shows that the physiologic signal aligns most consistently with objective, trained-annotator boundaries, and that subjective boundaries become more physiologically informative when they capture the same high-salience structural events. For this reason, we interpret the boundary results as primarily structure-driven, with perception contributing mainly through how accurately individuals detect those structural transitions.

### Music structure envelope's effect on coherence

Across both opera excerpts, coherence increases were time-locked to salient music transitions, with stronger and more consistent effects in Nabucco, mirroring.^9^ Nabucco contains clearer, more predictable boundary structure, and coherence peaks clustered around phrase-level timing (including ∼0.1 Hz, overlapping Mayer-wave-range dynamics).^25,28,36^ These temporal correspondences suggest that alignment is facilitated when changes in music unfold on timescales that resonate with intrinsic autonomic rhythms. Peaks were particularly evident around fortissimi passages, consistent with stronger structural salience and abrupt changes in musical energy.

In contrast, La Traviata exhibits more continuous variability and less sharply demarcated sections, which may reduce the stability of physiologic alignment.^15–17^ While such variability may enhance perceptual engagement, it appears less conducive to sustained autonomic synchronization. Together, these observations support the notion that boundary clarity, predictability, and the strength of music transitions play a central role in shaping entrainment.^28^

Methodologically, we matched Bernardi *et al*.’s participant demographics while extending the analysis using MIR-derived features and time–frequency coherence to capture fine-grained temporal dynamics. We focused on two excerpts to enable deeper physiologic and perception-based modelling rather than maximizing stimulus diversity. Because excerpts were presented in a fixed order, order effects cannot be excluded; however, the consistent pattern of stronger entrainment for the more structurally regular excerpt aligns with prior evidence that predictability and boundary salience support entrainment of biological rhythms.^22,23,28,37,38^ This convergence suggests that the observed differences are unlikely to be driven solely by presentation order. Future studies should counterbalance stimulus order to formally isolate stimulus-specific effects from potential presentation-order influences.

Surrogate testing further supported that observed coherence changes were linked to music structure rather than incidental physiologic fluctuations. Tempo emerged as a prominent driver of entrainment (*Table 1*), with robust coupling to blood pressure components in Nabucco, whereas La Traviata showed fewer robust effects, more of which consistently involved RR intervals. Exploratory chorister vs. non-chorister comparisons suggested broadly similar entrainment profiles across groups. The apparent respiration-related differences should be interpreted cautiously, given limited statistical power and may reflect respiration-mediated coupling during vocal music listening rather than training-specific autonomic mechanisms.^24,39,40^

The increased intra-individual coherence between physiology-to-physiology pairs during music vs. silence, along with increased inter-individual coherence during music, indicates that music influences autonomic signals. Its effect is seen in how the signals interact with one another and how it leads to synchronization between individuals, corroborating other findings in the field.^4,24,41–44^ Whether these entrainment effects persist beyond active listening remains to be tested in longitudinal and post-listening analyses. Confirming a sustained effect even after the auditory stimulus ends would corroborate the findings of papers investigating the long-term effect of music listening^45–47^ and show promise for the use of music for long-term cardiovascular therapies.

### Clinical and translational implications

These findings suggest a clinically relevant framework in which structured music can be used as a non-invasive indicator of autonomic fitness. Because physiologic entrainment tracked objective, salient music boundaries consistently across individuals, music with well-defined structural transitions could be used to elicit controlled, concomitant autonomic responses. Such responses may provide a dynamic biomarker of autonomic flexibility or cardiovascular plasticity, complementing established measures such as heart rate variability. Entrainment strength could be used to identify individuals who are more responsive to autonomic modulation through non-pharmacologic means, thereby helping to stratify those most likely to benefit from music-based interventions.

Beyond assessment, the ability to reliably engage the autonomic system through specific music transitions raises the possibility of using music as a controlled physiologic stimulus to promote periods of increased vagal tone, with potential implications for cardiovascular risk reduction. Repeat exposure to music that systematically engages autonomic responses could function as ‘exercise’ for the autonomic nervous system, allowing future studies to test whether short-term entrainment effects translate into long-term improvements in baseline autonomic state. Importantly, the consistent responses to objective music structure suggest that such approaches could be standardized while still allowing subsequent tailoring based on individual responsiveness. Together, these findings motivate future longitudinal and interventional studies examining whether music-induced autonomic engagement can be leveraged both as a diagnostic tool and as a therapeutic strategy for supporting cardiovascular and autonomic health.

## Conclusion

Music’s capacity to entrain physiology is not simply a matter of preference or expertise, but is grounded in the structural organization of the sound itself. By showing that well-defined phrase boundaries and predictable temporal environments elicit stronger autonomic synchronization to music structures, this study points to a concrete mechanism through which music can modulate cardiovascular and respiratory rhythms. These insights not only deepen our understanding of how perception and physiology interact, but also provide a framework for designing music therapies. By clarifying which music features are especially entraining—like the predictability and strength of boundaries—(such as for Nabucco) our results underscore music’s potential as a scalable, non-pharmacological tool for supporting autonomic regulation and cardiovascular health.

## Supplementary Material

qyag025_Supplementary_Data

## Data Availability

The data underlying this article will be made available via PhysioNet following de-identification and final dataset curation. A persistent identifier (DOI) will be assigned by PhysioNet at the time of release and will be added to the final version of the manuscript. Until public release, data is available from the corresponding author upon reasonable request. No third-party datasets were analysed.

## Lead author biography

Natalia Cotic is a PhD student in Biomedical Engineering whose research examines physiological and musical signal interactions within the HeartFM project. Her work focuses on computational modelling of heart rate variability, respiration, and blood pressure in response to performed and composed musical structures. She holds a Bachelor’s and Master’s degree in Biomedical Engineering from King’s College London, where her research centred on real-time convolutional neural network–based denoising of catheterization laboratory images.
