# Supplementary material for: Autonomic entrainment to music structure in Verdi opera
Source: Eur Heart J Imaging Methods Pract. 2026 Feb 10;4(1):qyag025. doi: 10.1093/ehjimp/qyag025 (PMC12968618; doi:10.1093/ehjimp/qyag025)
Supplement: qyag025_Supplementary_Data [file qyag025_supplementary_data.docx]

**Supplementary Material S1**

Following the pre-processing steps, the first analysis method involved calculating the coherence between the signals to validate the findings of (9). To quantify the dynamic coupling between physiological signals and musical features, we computed time--frequency coherence using the smoothed pseudo–Wigner–Ville distribution (SPWVD) (29,30). This approach provides high temporal and spectral resolution and is well-suited for resolving slow cardiovascular oscillations such as Mayer waves (~0.01Hz), which were a highlighted characteristic in (9).

Given a signal $x(t)$, its SPWVD is defined as:

1. $S_{xx}^{\text{SPWVD}}(t,f)=\iint A_{xx}(\tau,\nu)\text{ }\Phi(\tau,\nu)\text{ }e^{j2\pi(\nu t-\tau f)}\text{ }d\tau\text{ }d\nu$,

where $A_{xx}(\tau,\nu)$ is the **ambiguity function**:

1. $A_{xx}(\tau,\nu)=\int x\text{ }\left( t + \frac{\tau}{2} \right)\text{ }x^{*}\text{ }\left( t - \frac{\tau}{2} \right)\text{ }e^{-j2\pi\nu t}\text{ }dt$,

and $\Phi\left( \tau,\nu\right)$ is the **elliptical exponential kernel**,

1. $\Phi(\tau,\nu)=\exp[\text{ }-\pi(\lambda\text{ }\tau^{2}+(\nu/v_{0})^{2}+(\tau/\tau_{0})^{2})]$

parametrised by $\left( v_{0} , \tau_{0} , \lambda\right)$, which jointly control the degree of smoothing and the trade-off between time and frequency resolution. The parameter values were selected to prioritise the detection of physiologically relevant low-frequency dynamics by testing the results for different parameter pairings (31,32). The kernel was centred at (0,0) in the time-frequency domain and applied identically across all analyses to ensure comparability. This was possible as all signals had the same sampling frequency of 4Hz following interpolation.

Physiological modulations that were too slow to reflect responses to musical changes were filtered out. A physiological signal’s dynamic variability component was obtained by first estimating its slow trend using a low-pass Butterworth filter with a cut-off of ~0.03Hz. The filtered output (which contained the variations slower than ~0.03Hz) was then subtracted from the original signal. This produced a residual signal containing only fluctuations faster than 0.03Hz. These faster variations reflected beat-to-beat or breath-to-breath modulations rather than longer trends that would not reflect modulations caused by changes in the music. In the coherence analysis, it was these variability components of the signals that were used as inputs for the function used. Music-physiology comparisons showed how physiology entrained to the music during listening, while physiology-physiology comparisons showed how the body’s internal interactions are influenced by the music.

For each signal pair (two signals $x_{1}(t)$and $x_{2}(t)$), we computed the quadratic time-frequency coherence using the TFCspwv function (33).

1. $\gamma_{12}^{2}\left( t,f \right)=\frac{{\mid S_{12}^{\text{SPWVD}}\left( t,f \right)\mid}^{2}}{S_{11}^{\text{SPWVD}}\left( t,f \right)\text{ }S_{22}^{\text{SPWVD}}\left( t,f \right)}$

where S_11_ and S_22_ denote the individual SPWVDs of the two signals and S_12_ their cross-SPWVD, all evaluated using the same kernel $\Phi\left( \tau,\nu\right)$. The resulting three-dimensional coherence matrix $\gamma_{12}^{2}\left( t,f \right)$ quantifies the strength of coupling between the instantaneous spectral components of the signals over time. This method enabled us to identify coherence peaks associated with musical transitions.

Statistical significance was assessed using within-subject surrogate testing. Surrogates provided a participant-specific null distribution. Observed coherence values were compared to these distributions. The surrogate sets were created by concatenating an individual’s physiological data for the entire recording, excluding the track being analysed, and then shuffling the order (34,35). Length-matched segments were then randomly chosen, and the analysis was run with this surrogate data. For each of the 1000 iterations, one new randomly chosen surrogate segment was chosen at random. By using an individual’s own data, we accounted for the unique underlying patterns that each person exhibits. The distribution containing the real values were compared with the distributions comprised of the means of each individual’s surrogate results.

For each participant, we computed the difference between the observed coherence and the participant's surrogate mean. A t-test on these within-subject differences assessed whether observed coherence differed from the surrogate one. This allowed us to ascertain whether or not the trend observed in the real values could be attributed to the music being listened to. Bonferroni correction was applied to account for having 6 tests per music feature of a track, resulting in the new p-value being 0.008.

To identify if there is a clear influence on the coherence due to the music, we compared the music envelope (Hilbert transform of the audio signal) with the mean coherence envelope of all participants. The visual representation of this is seen in Figure 3, which is described further in the Results section.
